# State Estimation of Gas-Lifted Oil Well Using Nonlinear Filters

**DOI:** 10.3390/s22134875

**Published:** 2022-06-28

**Authors:** Ojonugwa Adukwu, Darci Odloak, Amir Muhammed Saad, Fuad Kassab Junior

**Affiliations:** 1 Department of Telecommunications and Control, University of Sao Paulo, Sao Paulo 05508-010, Brazil; fuad@lac.usp.br; 2 Department of Industrial and Production Engineering, Federal University of Technology Akure, Akure 340110, Nigeria; 3 Department of Chemical Engineering, University of Sao Paulo, Sao Paulo 05508-010, Brazil; odloak@usp.br; 4 Intelligent Techniques Laboratory, University of Sao Paulo, Sao Paulo 05508-010, Brazil; amir.saad@usp.br

**Keywords:** extended Kalman filter, gas lift, particle filter, unscented Kalman filter, sensor

## Abstract

The focus of this work is the extension of nonlinear state estimation methods to gas-lifted systems. The extended Kalman filter (EKF), unscented Kalman filter (UKF) and particle filter (PF) were used to estimate the nonlinear states. Brief descriptions of the filters were first presented starting from the linear Kalman filter. Hypothesis tests on the expectation of the residuals were performed to show how close to optimal the estimation methods are and it showed the UKF estimates to be slightly better than EKF while PF performs the worst. The PF has poor accuracy using residual visualisation, hypothesis test and the root mean squared error (RMSE) values of the residuals. The gas-lifted system exhibits casing heading instability where the states show oscillatory behaviour depending on the value of the input but the results here do not change in a known way for each filter as the input is changed from the non-oscillatory region to the oscillatory region. Therefore, for this noise distribution and model assumption, either the EKF or UKF can be used for nonlinear state estimation with UKF better preferred if computational cost is not considered when control solutions are used in gas-lifted system.

## 1. Introduction

Gas-lifted systems are usually situated in harsh environments or deep below the sea surface for sensors to produce reliable measurements. This is due to the difficulty encountered when deploying the sensors in the location required to provide good measurement, excess heat effect on the sensors, and reaction between the sensors and the harsh environments among other reasons.

Most control solutions in gas-lifted systems rely on these sensor measurements, hence the states or variables used in the controllers are unreliable. This is the case in [1,2] where a control solution was used to remove casing heading instability assuming all measurements were available and reliable. However, in [3,4,5] top-side measurements were used by the observers and Kalman filters to estimate the states for the controller.

The performances of these filters on gas-lifted systems vary due to the dependence of the model on the underlying assumptions used at the modelling stage, in addition to the noise assumption. In the linear case, the Kalman filter is the optimal estimation method used [6,7]. A gas-lifted system is inherently nonlinear, and if it is used in this form, nonlinear filters such as the extended Kalman filter (EKF) [8], unscented Kalman filter (UKF) [9,10] or particle filter (PF) [11] among other nonlinear state estimation methods should be used. These nonlinear state estimation methods have been applied successfully in many fields such as navigation [12], robot localisation [13] and fault detection in chemical process [14], but they have not found much application in gas-lifted systems. A close application to gas-lifted systems are the use of the EKF for leak detection in pipes (which may not necessarily be gas lift pipes) [15] and the UKF with linear model predictive control (MPC) for optimisation of gas-lifted systems [16].

This limited application of nonlinear filters in gas-lifted systems is due to the fact that linearising the system about an operating point and applying the linear Kalman filter is usually sufficient in most control applications. However, as the demand for optimal operation of gas-lifted systems is increasing, using the nonlinear filters makes it possible to estimate the states at various points, hence making it easier to approach these demands as much as possible. The choice of filter type to use in state estimation depends on the accuracy requirements, the complexity of the filter, the computational demand, the speed of convergence and the degree of linearity of the system among other factors.

A hybrid approach to optimisation of gas-lifted systems proposed in [17] reduces the steady state wait-time associated with static real-time optimisation (RTO). This hybrid approach uses model adaptation that involves parameter updates using dynamic models while optimisation takes place using static nonlinear models. To meet up with the model adaptation, speed of convergence is the most important consideration for filter selection. Faster filters such as that proposed in [18] which uses a direct approach to parameter estimation are used to decrease the convergence time of the parameter estimation. When the sensor measurements are unreliable, this hybrid approach can be implemented at a more optimal level using both state and parameter estimation approach like that presented in [19].

In this article, we discuss and apply the EKF, UKF and PF to gas-lifted systems and examine the performances of these filters on the system. We compare the performances of the filters on the system by examining the residuals and performing hypothesis tests on the residuals. This research provides a basis to select a nonlinear filter based on estimation accuracy and computational demand when the gas-lifted system is to be operated with nonlinear models. The outputs from these nonlinear filters in gas-lifted systems are useful for decision making in areas of control application such as fault detection and diagnosis, casing heading instability removal and general optimal operation of the system.

This paper is organized as follows: Section 1 introduces the paper. Section 2 discusses the state estimation methods considering the linear Kalman filter before discussing the main nonlinear filters of EKF, UKF and PF. Section 3 discusses gas-lifted systems and presents the differential algebraic equations (DAEs) that describe them. Section 4 presents the results and discussion which is the filter performances and Section 5 concludes the paper.

## 2. Materials and Methods—State Estimation

We present key features of various estimation techniques before comparing their performances. We first present the linear Kalman filter upon which the nonlinear filters that we want to apply are built. In all the filter types presented, the estimation processes follow the same pattern of prediction and update. What differentiates the estimation processes is the method of prediction and update. Additionally, within the same filter type, these procedures can differ depending on the computational demand, accuracy or other considerations.

### 2.1. Kalman Filter (KF)

The Kalman filter (KF), introduced in 1960, estimates states of linear system whose model is not exactly known and the inaccuracy represented by the model noise is given in (1), with the noise itself as given in (2) [20]:

(1a) $$\begin{array}{cc} \; & x_{k}=Ax_{k-1}+Bu_{k}+w_{k}, \end{array}$$

(1b) $$\begin{array}{cc} \; & y_{k}=Cx_{k}+v_{k} \end{array}$$

(2) w≈N(0,R),v≈N(0,Q)

where *w* and *v* are the state and output noise respectively which are assumed to be white, uncorrelated and follow gaussian distribution of zero mean and non-zero covariance. *A*, *B* and *C* are state transition matrix, input matrix and output matrix respectively.

The Kalman filter is an optimal estimator for a linear system even if *w* and *v* are still not Gaussian. The Kalman filter combines information from system prediction and measurement to obtain the best estimates of the system by multiplying their probability density functions (PDFs). The gain is selected based on which provides more reliable information between the state estimates up to a given time instant and the measurement at the given time instant which are indicated by the covariance. This is done through an iterative process that involves the prediction stage and correction stage as described below:

Prediction:

(3a) $$\begin{array}{cc} \; & {\hat{x}}_{k}^{-}=A{\hat{x}}_{k-1}+Bu_{k}, \end{array}$$

(3b) $$\begin{array}{cc} \; & P_{k}^{-}=AP_{k-1}A^{T}+Q \end{array}$$

Correction:

(4a) $$\begin{array}{cc} \; & K_{k}=P_{k}^{-}C^{T}{\left(CP_{k}^{-}C^{T}+R\right)}^{-1}, \end{array}$$

(4b) $$\begin{array}{cc} \; & {\hat{x}}_{k}^{+}={\hat{x}}_{k}^{-}+K_{k}\left(y_{k}-C\hat{x}\right), \end{array}$$

(4c) $$\begin{array}{cc} \; & P_{k}^{+}=\left(I-K_{k}C\right)P_{K}^{-} \end{array}$$

*P*, *Q* and *R* are the state error covariance, state noise covariance and measurement noise covariance respectively while *K* is the Kalman gain. The initial values for the estimated state and error covariance are [21]:

(5) $$\begin{array}{cc} \; & {\hat{x}}_{0}^{+}=E\left(x_{0}\right),\\ \; & P_{0}^{+}=E\left[\left(x_{0}-{\hat{x}}_{0}^{+}\right){\left(x_{0}-{\hat{x}}_{0}^{+}\right)}^{T}\right] \end{array}$$

where *E* implies expectation.

At the prediction stage, the states are predicted based on the system model in (1a). Then the state error covariance matrix is obtained according to (3b), giving ${\hat{x}}_{k}^{-}$ (a priori state) and $P_{k}^{-}$ (a priori state error covariance) respectively. These are used to compute the Kalman gain (K) at the correction stage. The estimated state is obtained based on the available measurements and the state prediction. The error covariance of the state estimates is then calculated from the estimated states giving ${\hat{x}}_{k}^{+}$ and $P_{k}^{+}$ which are the posterior states and error covariances respectively.

### 2.2. Nonlinear Filters

The nonlinear filters are built on the ideas of the Kalman filter. The extended Kalman filter (EKF) is used for low nonlinear systems with Gaussian noise distribution. It computes the Jacobian matrices of the state transition function in (6a) with respect to the state and the state noise at previous state estimates to obtain *A* and *W* respectively. Similarly, it computes the Jacobian matrices of the output function in (6b) with respect to states and the output noise to obtain matrices *C* and *V* respectively.

(6a) $$\begin{array}{cc} \; & x_{k}=f_{k-1}\left(x_{k-1},u_{k-1},w_{k}\right), \end{array}$$

(6b) $$\begin{array}{cc} \; & y_{k}=g_{k}\left(x_{k},v_{k}\right) \end{array}$$

The prediction and update procedure for the EKF is then given in (7) and (8) respectively [21].

Prediction:

(7a) $$\begin{array}{cc} \; & {\hat{x}}_{k}^{-}=f_{k-1}\left({\hat{x}}_{k-1}^{+},u_{k-1},0\right), \end{array}$$

(7b) $$\begin{array}{cc} \; & P_{k}^{-}=A_{k-1}P_{k-1}A_{k-1}^{T}+W_{k-1}Q_{k-1}W_{k-1}^{T} \end{array}$$

Correction:

(8a) $$\begin{array}{cc} \; & K_{k}=P_{k}^{-}C_{k}^{T}{\left(C_{k}P_{k}^{-}C_{k}^{T}+V_{k}R_{k}V_{k}^{T}\right)}^{-1}, \end{array}$$

(8b) $$\begin{array}{cc} \; & {\hat{x}}_{k}^{+}={\hat{x}}_{k}^{-}+K_{k}(y_{k}-g_{k}\left({\hat{x}}_{k}^{-},0\right), \end{array}$$

(8c) $$\begin{array}{cc} \; & P_{k}^{+}=\left(I-K_{k}C\right)P_{K}^{-} \end{array}$$

Other variants of the EKF exist that minimise the linearisation error inherent in the EKF. These variants are called higher order EKF as they consider higher order terms in the Taylor series linearisation [22].

For highly nonlinear systems with Gaussian noise distribution, the unscented Kalman filter (UKF) is a better estimator than the extended Kalman filter (UKF). The UKF represents the system state space by a few carefully selected sigma points and uses the unscented transform (UT) to obtain the statistics of the estimated states. The prediction stage in UKF is a three-step process that involves forming the matrix of sigma points, propagating the sigma points through the nonlinear model and obtaining the estimates for the mean and covariance through a third order linearisation of the nonlinear system. While the correction step involves transforming the sigma points into measurement space using (6b) to obtain $y_{t}$ and computing the transformed mean and covariance of the estimated states.The use of three terms in the linearisation improves the accuracy, hence minimises the linearisation error in the UKF.

When the system is nonlinear and the state distribution is non-Gaussian, the UKF becomes insufficient for the state estimation hence particle filter is used. The PF represents the system states with particles similar to sigma points in UKF although the particles are randomly selected not using the algorithm as in UKF. The estimate is determined by the probability that the state takes a certain value hence the state transition function, the state estimates and other variables are expressed as PDF. The final estimate is then obtained from the PDF using any method that is desired such as mean, expectation, states with maximum weight, etc. The PF has prediction, correction and re-sampling stages. At the prediction stage, state hypotheses or particles are generated from the initial states of the system and propagated through the state transition function. At the correction stage, conditional PDF of the measurement, $p(z_{k}|x_{k})$ is computed based on the measurement function in (6b) and the knowledge of the PDF of the noise $\left(v_{k}\right)$. The PDF of the estimated states is thereafter obtained. At the resampling state, new particles are selected again based on the weights. The weights are used to compute a value that shows if the current particles still represent the state distribution accurately enough. This value, called effective number of particles and denoted as $N_{eff}$, is given in (9) as:

(9) $$N_{eff}=\frac{1}{\sum_{i=1}^{N}{\left(W_{k}^{i}\right)}^{2}}$$

Since the weight *w* is normalised to sum to 1, the more particles that still contribute to the distribution (particles with reasonable weights), the more the value of $N_{eff}$. Hence a threshold is set which when $N_{eff}$ falls below, resampling takes place. Resampling can also take place at a fixed time interval or using a threshold defined based on the inverse of the particle weights. In [23], however, it is shown that using the $N_{eff}$ reduces the number of resampling steps compared to the other methods described above.

Table 1 presents a summary of the major estimation types discussed briefly. It is seen there that the complexity of the estimation type varies from the very simple linear Kalman filter to the very complicated particle filter which also shows the variation of the performance.

## 3. The Gas-Lifted System

**Figure 1:**
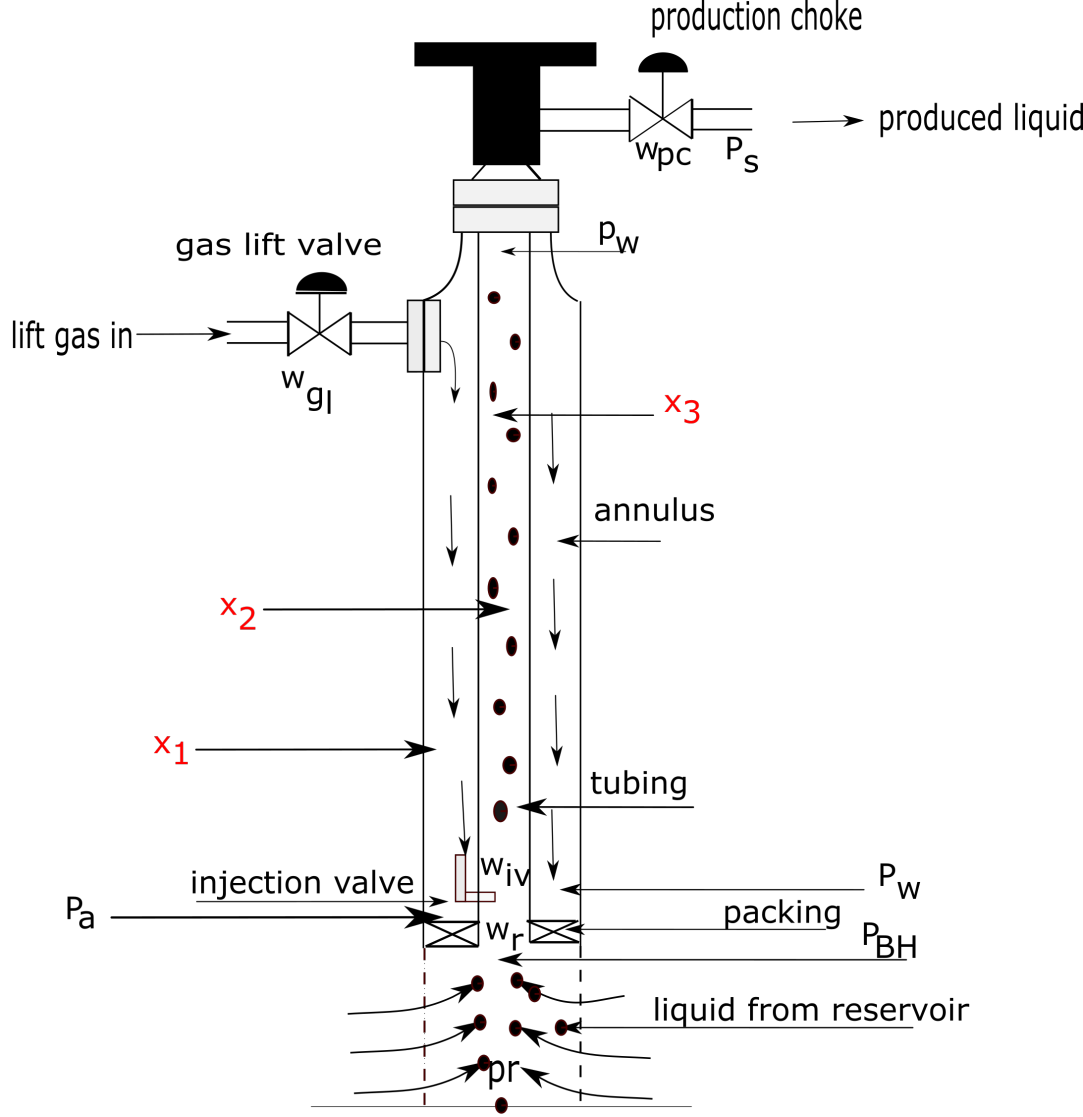
A single well gas-lifted system showing the states in red. These states are difficult for the sensors to measure accurately hence must be estimated by filters using the information from the sensors before being used for any control solution.

The gas-lifted system is one of the artificial lift methods employed when the natural energy for lifting hydrocarbons from the reservoir into the production platforms becomes insufficient. Figure 1 is a schematic of a gas-lifted system. The states are the mass of gas in the annulus, the mass of gas in tubing and the mass of oil in tubing indicated as $x_{1}$, $x_{2}$ and $x_{3}$ respectively. A gas-lifted system sometimes goes to a depth of several hundreds of metres below sea level making it difficult for sensors to provide accurate measurements of the states.

The key features of the system are the two volumes which are the annulus (that holds $x_{1}$) and tubing (that holds $x_{2}$ and $x_{3}$). The reservoir pressure ($P_{r}$) provides the natural energy for lifting the crude up to the storage tank. Lift gas enters through the gas lift valve and out through the production choke while the injection valve connects the annulus and the tubing. All variables in gas-lifted system are positive, hence a gas-lifted system is a positive system.

### 3.1. Gas-Lifted System Models

Gas-lifted system models represented by partial differential equations like those in [24,25] more accurately describe the system but are more complex. As noted in the introduction Section 1, the models of gas-lifted systems depend on the underlying assumption made. The more complex models are, the more difficult to use, hence trade-offs are usually made between model accuracy and ease of use for control application. For our estimation problem here, we use a slight modification of the models presented in [26] which were verified in OLGA software to be very close to the real system. These models are presented below:

The mass (differential equations):

(10) $$\begin{array}{cc} \; & \frac{dx_{1}}{dt}=w_{gl}-w_{iv} \end{array}$$

(11) $$\begin{array}{cc} \; & \frac{dx_{2}}{dt}=w_{iv}+w_{rg}-w_{pg} \end{array}$$

(12) $$\begin{array}{cc} \; & \frac{dx_{3}}{dt}=w_{ro}-w_{po} \end{array}$$

The flowrate:

(13) $$\begin{array}{cc} \; & w_{iv}=C_{iv}\sqrt{max(0,\rho_{a}\left(P_{a}-P_{w}\right))} \end{array}$$

(14) $$\begin{array}{cc} \; & w_{pc}=C_{pc}\sqrt{max(0,\rho_{t}\left(P_{wh}-P_{s}\right))}f\left(u\right) \end{array}$$

(15) $$\begin{array}{cc} \; & w_{ro}=C_{iv}\sqrt{max(0,\rho_{0}\left(P_{r}-P_{bh}\right))} \end{array}$$

(16) $$\begin{array}{cc} \; & w_{pg}=\left(\frac{x_{2}}{x_{2}+x_{3}}\right)w_{pc} \end{array}$$

(17) $$\begin{array}{cc} \; & w_{po}=\left(\frac{x_{3}}{x_{2}+x_{3}}\right)w_{pc} \end{array}$$

(18) $$\begin{array}{cc} \; & w_{rg}=GORw_{ro} \end{array}$$

The pressure:

(19) $$\begin{array}{cc} \; & P_{a}=\left(\frac{T_{a}R}{V_{a}M_{w}}+\frac{gL_{a}}{V_{a}}\right)x_{1} \end{array}$$

(20) $$\begin{array}{cc} \; & P_{wh}=\frac{T_{t}R}{M_{w}}\left(\frac{x_{2}}{V_{t}-\frac{x_{3}}{\rho_{0}}}\right) \end{array}$$

(21) $$\begin{array}{cc} \; & P_{w}=P_{wh}+\left(x_{2}+x_{3}\right)\frac{g}{A_{t}} \end{array}$$

(22) $$\begin{array}{cc} \; & P_{bh}=P_{w}+\rho_{0}gH_{bh} \end{array}$$

The density:

(23) $$\begin{array}{cc} \; & \rho_{a}=\frac{M_{w}}{T_{a}R}P_{a} \end{array}$$

(24) $$\begin{array}{cc} \; & \rho_{a}=\frac{x_{2}+x_{3}}{V_{t}} \end{array}$$

(25) $$\begin{array}{cc} \; & f\left(u\right)=50^{u-1} \end{array}$$

The mass equations give the differential equations while the pressure, flow rate and density give the algebraic equations, hence system model is a differential algebraic equation (DAE) of the form (26).

(26a) $$\begin{array}{cc} \; & ẋ=f(x,z,u), \end{array}$$

(26b) $$\begin{array}{cc} \; & 0=g(x,z,u), \end{array}$$

Table 2 lists the symbols, definitions and units of the variables used in the models while Table 3 lists the constants, definitions, units and values used in this article. Equation (25) shows how the input u, which is the percentage valve opening is applied to the flow rate through the production choke to prevent zero flow rate in this case. The max in the flow rate equations ensures that the upstream pressure is bigger than the downstream pressure else a flowrate of zero is produced to ensure a non-negative flowrate. $V_{a}$ and $V_{t}$ are annulus and tubing volumes respectively and are calculated from their respective areas and lengths.

### 3.2. Gas-Lifted Input and Measurements

The input here is the percentage valve opening that controls the flow rate of produced fluid through the production choke. In other cases, the flow rate of lift gas into the annulus is also considered as input but we fix it here with a regulatory controller. The measurements are annulus pressure ($P_{a}$), wellhead pressure ($P_{wh}$) and mixture density ($\rho_{t}$) which are readily available from the sensors and are reliable [6]. The states as stated earlier are the masses presented in Equations (10)–(12).

## 4. Results and Discussion—Filters Performances on Gas-Lifted Systems

In this section, we compare the performances of the three nonlinear filters on the gas-lifted system. The assumed distribution is Gaussian for all three filters. The initial condition, $x_{0}$ = [2300 750 5800] kg is the same for all three filters and the input is fixed at u=0.6 throughout for all three filters. The state covariance, $P_{0}=diag$([100 10 1000]), the state noise, Q=diag([100 16 160]) and the measurement noise, R=diag([1000 1000 40]) are the same for all three filters. The sampling time is 60 s (1 min) for all three filters and the system is simulated over 150 samples (2.5 h). For the UKF, $\alpha =10^{-3}$, β=2 and κ=0. The PF uses maximum weight to obtain the state estimates from the posterior distribution since the weight reflects the probability that the true state is the given particle (i.e., the states with higher weight have a higher chance of being the true state of the system). The threshold for triggering resampling is set to 0.8 to quickly remove particles that are not contributing significantly to the distribution while the sampling method is residual. All the simulations were done in MATLAB version R2021a [27]. Euler and ODE15S were used to solve state trajectories of the differential algebraic equation (DAE).

### 4.1. States and Residuals Visualisation

**Figure 2:**
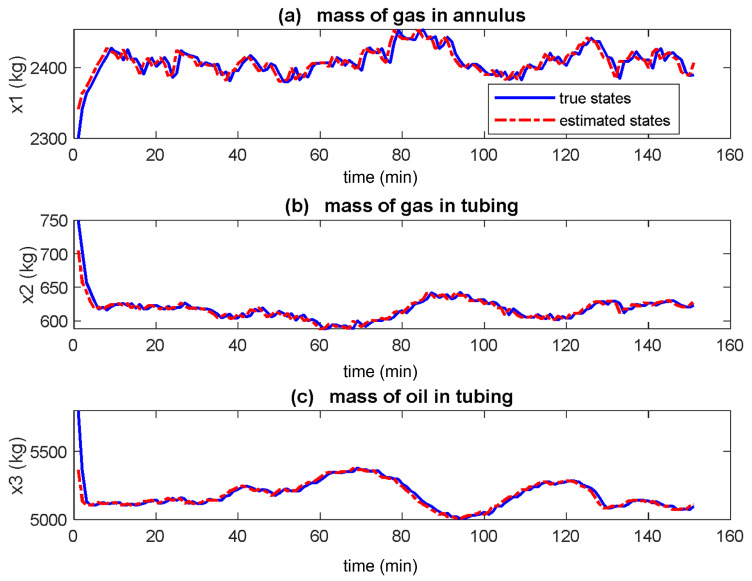
True and estimated states of gas-lifted systems using the extended Kalman filter (EKF). All three states estimates (red dash-dotted lines) converge to the true states (blue solid line) about the same time. These states are the mass of gas in annulus, the mass of gas in tubing and the mass of oil in tubing respectively.

**Figure 3:**
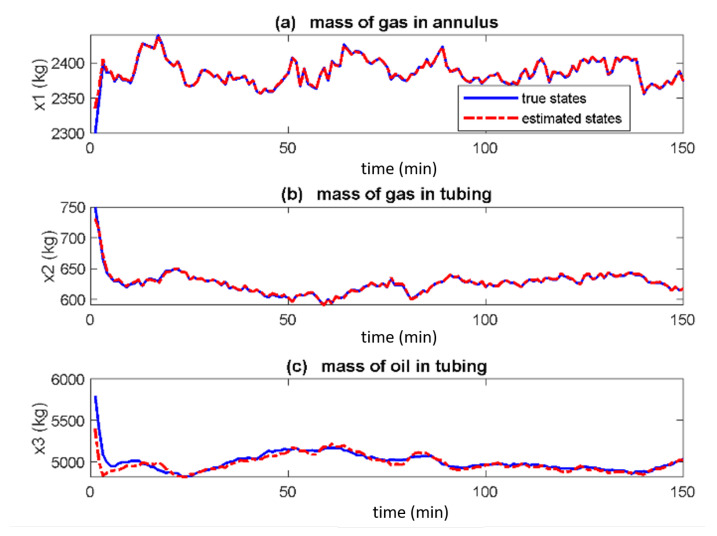
True and estimated states of gas-lifted systems using the unscented Kalman filter (UKF). The estimated states converge at different times with $x_{2}$ being the fastest and $x_{3}$ slowest.

**Figure 4:**
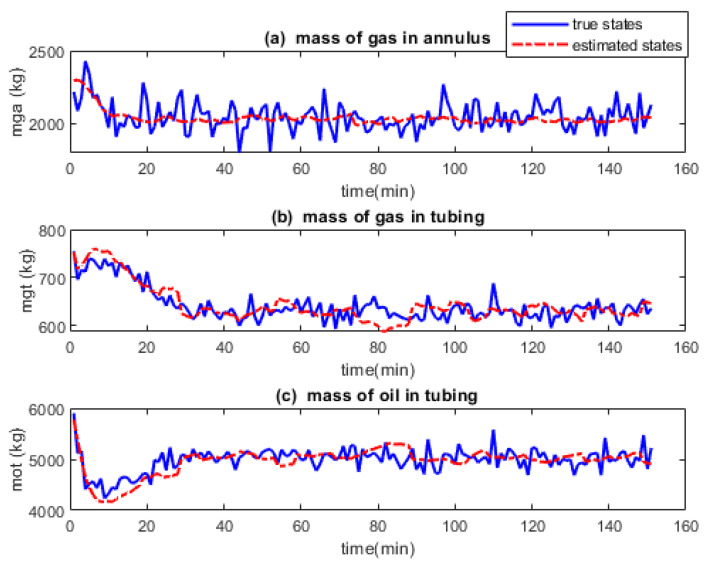
True and estimated states of gas-lifted system using a particle filter (PF). The estimated states do not converge to the true states exactly due to the particle sampling techniques used by PF.

Figure 2, Figure 3 and Figure 4 show the true and estimated states for the EKF, UKF and PF respectively. In Figure 2 the estimated states (red dash, dotted) converge to the actual states (blue solid) in under 10 min for all three states. The estimated states converge to the true states in Figure 3 too but at different times with $x_{2}$ fastest whereas $x_{3}$ being the slowest. Figure 4 shows that estimated states track the actual states poorly.

The effect of random sampling of the states into particles and obtaining the state estimates from the posterior distribution is seen in the poor tracking performance of the PF. While the UKF also samples the states before using the unscented transformation to obtain the anterior state statistics, the sampling here is carefully and deterministically done hence the UKF tracks better than PF. Unlike in the case of EKF and UKF where each state is propagated through the state transition function and is estimated individually, the PF propagates states hypotheses. The consequence of this is that the actual state is not being properly tracked like that of the EKF and UKF. The result is not better when the state estimate is obtained from the posterior distribution by using the mean of the particles despite that the true state is believed to be around the mean of the 3000 particles.

From Figure 2 and Figure 3, the UKF tracks better than EKF due to the use of three term approximation of the Taylor series of the nonlinear system by the UKF while EKF uses two terms. Hence based on the visualisation of the true and estimated states, the UKF performs better whereas the PF performs least. This result is different from the one performed on mechanical system in [12] where the EKF performed poorest while the performances of the UKF and PF were similar. This justifies the extension of these methods to gas-lifted systems as the performance of the filters depends also on the system whose states are estimated in addition to the noise distribution.

**Figure 5:**
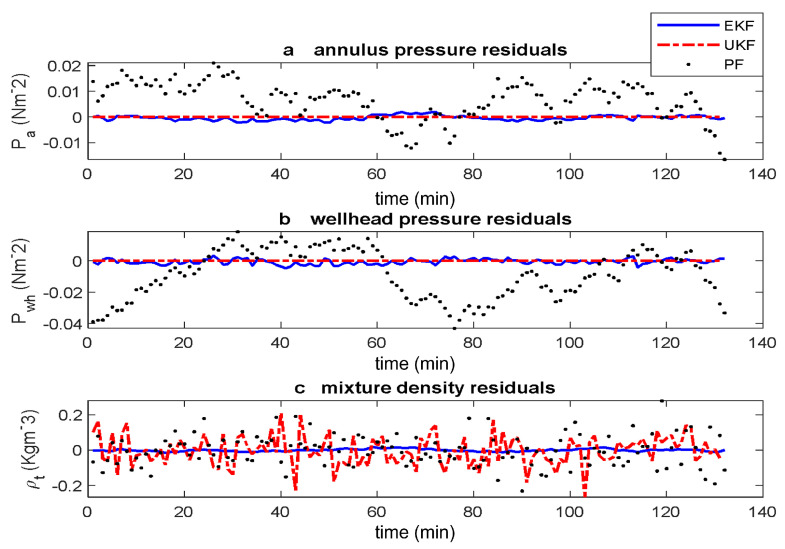
Estimated states full residuals of gas-lifted system using EKF, UKF and PF. The residuals from estimating using PF is the poorest for the three measurements.

The initial slow convergence of the states means that the residuals have a higher magnitude for the first few state estimates. We remove this transient part and show in Figure 5, the residuals. In all three measurements, the residuals from the PF is larger than the EKF and the UKF reaching a value of $2.1\times 10^{5}$, $-1.8\times 10^{5}$ and $1.8\times 10^{1}$ for $P_{a}$, $P_{wh}$ and $\rho_{t}$ respectively. The UKF has a smaller residual than the EKF for $P_{a}$ and $P_{wh}$ but bigger for $\rho_{t}$ indicating that the UKF still has the best estimation performance by residual visualisation.

**Figure 6:**
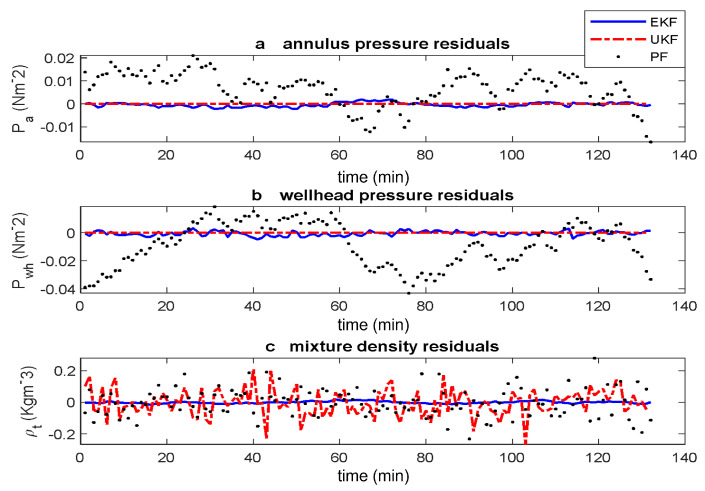
Estimated states normalised residuals of gas-lifted systems using EKF, UKF and PF. The normalised residuals are obtained from the residuals by dividing each residual by the corresponding steady state value.

We examine the residuals further by first considering the normalised residuals. The normalised residuals are obtained by dividing the residuals by the steady state values of the actual measurements given as $y_{True}$ = [$P_{a}$$P_{wh}$$\rho_{t}$] = [9,930,500 N m${}^{-2}$ 4,678,800 N m${}^{-2}$ 71.9316 kg m${}^{-3}$]. Figure 6 shows the normalised residual plots. For each output, the residuals resulting from the EKF (blue solid line), UKF (red dash-dotted line) and PF (blue dotted line) are compared.

We observe from Figure 6 that normalised residual has a very low value, especially for the EKF and the UKF. On zooming in on Figure 6 at steady state, these values are of the order of $1\times 10^{-3}$ for UKF and $3\times 10^{-3}$ for the EKF but about 0.2 and −0.2 for PF when the residual is the mixture density. This shows that the estimates are better in the EKF and UKF than in PF with the UKF still the best. Additionally, for the three measurements, the distribution of the residuals around the zero line is poor for the PF except in the case of $\rho_{t}$ in Figure 6c. Furthermore, the distribution of the residual for the EKF and UKF are however even around the zero line including in Figure 6c where the residual for the UKF is large. The more even the distribution of the residuals around the zero line, the better the estimated states.

The above estimation was performed at a fixed value of u=0.6 which is in the stable region. The performance of the filters depends on the degree of linearity of the system and the noise distribution. The gas lift system is seen to exhibit different behaviour as input increases from 0 to 1. We therefore, perform statistical tests on the residuals. The first check is to see if the residuals of the filter outputs follow the Gaussian distribution of zero mean and non-zero variance. This we obtain from the expectation test on the residual. Next, we examine the RMSE for the residuals of gas lift system at three different inputs: u=0.60, u=0.75 and u=0.90. These inputs correspond to the system in the stable region, the system sliding into the unstable region and the system fully into the casing heading instability region respectively.

### 4.2. Statistical Tests

Two tests were performed on the residual here: the expected value test to examine the shape of the residual distribution and the root mean square error to show how close the estimates are to the true states.

#### 4.2.1. Expected Value Test

Since it is impossible to perform an infinite number of experiments to determine if the mean of the residual is close to zero, we use the expected value test to infer the centre of the residual distributions and with hypothesis tests, we check the mean of the entire residual distribution. The residual (innovation since it is stochastic) is obtained as the difference between the actual measurements from sensors and the outputs computed using the output function in (6b) with the states being the estimated states. The residual that is more evenly distributed around zero produces a distribution that is close to normal and increases the accuracy of estimation. Non-normality of residual does not exactly translate into poor estimation, especially in the case of nonlinear states with many samples, however, it helps to compare the performance of the estimation methods. A total of 151 samples were used for each filter simulation and the mean value for the residuals is computed using (27).

(27) $$\bar{r}\left(k\right)=\frac{1}{N}\sum_{i=1}^{N}r_{i}\left(k\right)$$

where *r* is the residual *k* is the measurement index corresponding to $P_{a}$, $P_{wh}$ and $\rho_{t}$, *N* is the number of samples which is 151 here and $\bar{r}$ is the mean of the residuals.

The hypothesis test is conducted on the computed mean of the residuals for the three measurements and Table 4 shows the results for the nine residuals. A checkmark “√” represents the true hypothesis which is that the residual comes from a normal distribution while an “X” represents an alternative hypothesis which is that the residual does not come from a distribution that is normal. The corresponding *p*-values are also provided in the table.

Optimal estimation is associated with a linear Kalman filter where the state and the measurement functions are linear and the noise is Gaussian. The effect of this is that when the state with Gaussian distribution is estimated, the residual (innovation) is Gaussian. This is not the case with other filters whose models are nonlinear. We therefore use the hypothesis test on the residual to see how close the nonlinear filter residuals are to being Gaussian. As seen in Table 4, the entries for most of the hypothesis tests indicate that the residuals do not come from a distribution that is Gaussian except for the residual $\rho_{t}$ for both EKF and UKF. The acceptance of the true hypothesis in these two isolated cases is not enough to draw the conclusion that the residual of the mixture density is normal when estimated with EKF and UKF as this might have happened by chance.

The *p*-values that indicate the chance that the true hypothesis holds are also provided and they indicate that the EKF and UKF have a higher chance of having residuals that are normal since they have higher *p*-values than the PF. The *p*-values of the UKF are better (bigger) than that of EKF except for $P_{wh}$. The PF has the worst performance as the *p*-values are smaller than those of EKF and UKF. Both residual visualisation and the expected value test indicate that UKF is slightly more accurate than EKF with PF having the lowest estimation accuracy of the three filters for this system.

#### 4.2.2. Root Mean Square Error

The RMSE for the residual is the most common error metric for testing the accuracy of the filters [8,12,28]. We examine the RMSE for the filters under three different input conditions to see if the accuracy of the filter depends noticeably on the effect of the casing heading instability. Casing heading instability results from the oscillatory behaviour of system depending on the input value. These inputs are u=0.6, u=0.75 and u=0.9 which correspond to the stable region, the region going into casing heading instability and the region inside casing heading instability respectively. Table 5 shows the hypothesis test and the RMSE for the three filters for u=0.6, u=7.5 and u=0.9 respectively.

It can be observed from Table 5 for all the three input values, the hypotheses tests are the same except for the $P_{a}$, which changed to true hypothesis when 0.9 while it is the alternative hypothesis for u=0.60 and u=0.75. Again this might have happened by chance. While the RMSE of $P_{a}$ residuals resulting from estimating with EKF increases from $9.1\times 10^{3}$ to $14\times 10^{3}$ and $26\times 10^{3}$, for the same EKF, the $P_{wh}$ residual increases from $7.8\times 10^{3}$ to $7.9\times 10^{3}$ and then decreased to $7.6\times 10^{3}$ for u=0.6, u=0.75 and u=0.9 respectively. The RMSE of the $\rho_{t}$ residual for estimation using EKF increased from 0.52 to 0.97 to 2.1. The RMSE for estimates using the UKF does not show much variation in values as *u* is changed into the unstable region. The estimation using the PF behaves similar to the UKF as the RMSE for $P_{a}$ increases from $6.4\times 10^{4}$ to $7.1\times 10^{4}$ to $30\times 10^{4}$.

This shows that there is no defined behaviour of the estimation accuracy of the nonlinear filters as input changes from the non-oscillatory region to the oscillatory region. However, using UKF still shows better prospects as it appears to have the least of the change in RMSE when a gas-lifted system is operated within these input ranges. Additionally, note that the best indicator of the accuracy of the measurement is the value of the RMSE of the residuals and residual visualisation. The statistical test does not significantly affect the acceptance of the estimation accuracy considering that the states are nonlinear. This will be different if the states are linear and the noise distribution is Gaussian where having a normally distributed residual gives us confidence in the degree of accuracy of our estimation. Additionally, care should be taken to interpret X in Table 4 and Table 5 as a failure of the null hypothesis test and not as products (multiplication).

**Figure 7:**
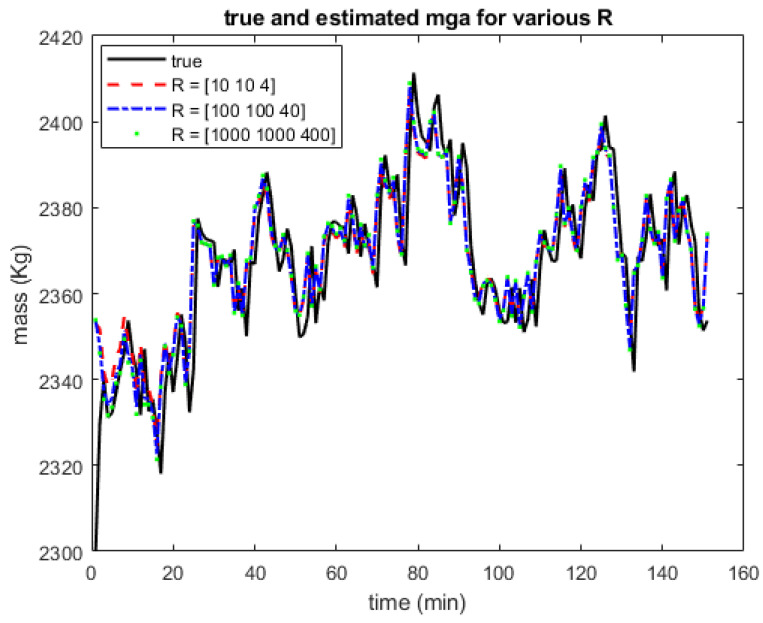
Estimated states and actual states for various values of *R*. The estimates did not change significantly with the values of *R*.

The estimation accuracy depends on the proper selection of the matrices *P*, *Q* and *R* as described in [8]. We examine how the change in *R* affects the filter performance using the EKF on $x_{1}$ (mass of gas in annulus). Figure 7 shows the true and estimated states for *R* = [10 10 4], [100 100 40] and [1000 1000 400] respectively. Figure 7 shows that there is no significant difference in the estimates for these different values of *R*. A minor difference exists during the transient state and during a major change in the direction of the graph. Similar results were obtained when the different filters were used with other states. This result indicates that for the gas-lifted system where we selected *P*, *Q* and *R* arbitrarily, the previous results were not affected significantly by these choices.

## 5. Conclusions

It is shown here that the gas lift system’s nonlinear states can be estimated directly using the EKF, UKF or PF without the need to linearise the system and apply a linear Kalman filter. Based on the noise assumption here, the UKF performs slightly better than the EKF by examining the RMSE for the residuals, visualising the states and examining the residual using the hypothesis test. This is because the UKF uses three term approximation of the Taylor series while the EKF uses one term. The additional terms improve the accuracy of the UKF over the EKF for the gas lift system. The performance of the PF is the worst. However, with the computational advantage of the EKF, the states of the gas lift system can be estimated using the EKF since there is just a small difference in performance between using it and the UKF. Hence for nonlinear control applications such as casing-heading instability, fault detection and diagnosis and general optimal operation of gas lifted systems, with Gaussian noise, the EKF can be used for state estimation.

The comparison here is based on the estimation error and does not consider explicitly the speed of convergence of the estimates to the true states because sampling times are larger in gas lift systems than in electrical and mechanical systems. Further works should consider both estimation error and the speed of convergence considering the importance of speed of convergence in parameter estimation for the hybrid optimisation in gas lift network.

## Figures and Tables

**Table 1:** Summary of the main estimation methods.

Estimator Type	System Type	Key Method Used	Complexity
Kalman Filter	Linear with Gaussian noise	Multiplication of the covariances	Very simple
Extended Kalman filter	Nonlinear with Gaussian noise	Linearisation/Kalman filter or partial derivative with nonlinear state transition function	Simple
Unscented Kalman Filter	Highly nonlinear with Gaussian noise	Selection of sigma points and utilisation of UT	Complex
Particle Filter	Highly nonlinear with non-Gaussian noise	Propagation of randomly selected particles from the initial states/use Bayesian approach for state estimates	Very complex

**Table 2:** Lists of symbols, definitions and units of the variables used in the models.

Variable	Definition	Unit
$w_{gl}$	Gas flowrate into the annulus	kg $s^{-1}$
$w_{iv}$	Gas flowrate from annulus into tubing	kg $s^{-1}$
$w_{rg}$	Gas flowrate from reservoir into tubing	kg $s^{-1}$
$w_{pg}$	Gas flowrate through the choke	kg $s^{-1}$
$w_{ro}$	Oil flowrate from reservoir into tubing	kg $s^{-1}$
$w_{po}$	Oil flowrate through the choke	kg $s^{-1}$
$w_{pc}$	Mixture flowrate through the choke	kg $s^{-1}$
$P_{a}$	Annulus pressure	N m${}^{-2}$
$P_{wh}$	Wellhead pressure	N m${}^{-2}$
$P_{w}$	Tubing pressure	N m${}^{-2}$
$P_{bh}$	Bottomhole pressure	N m${}^{-2}$
$\rho_{a}$	Annulus gas density	kg m${}^{-3}$
$\rho_{t}$	Tubing mixture density	kg m${}^{-3}$

**Table 3:** List of the constants, definitions, units and values used in this article.

Parameter	Definition	Unit	Value
$H_{a}$	Height of annulus	m	1500
$D_{a}$	Diameter of annulus	m	0.189
$H_{t}$	Height of tubing	m	1500
$D_{t}$	Diameter of tubing	m	0.121
$H_{bh}$	Height of bottomhole	m	500
$D_{bh}$	Diameter of bottomhole	m	0.121
$C_{r}$	Reservoir valve coefficient	m${}^{3}\;h^{-1}$	2.6 × 10^−4^
$C_{iv}$	Injection valve coefficient	m${}^{3}\;h^{-1}$	10^−4^
$C_{pc}$	Choke valve coefficient	m${}^{3}\;h^{-1}$	2 × 10^−3^
$\rho_{0}$	Reservoir oil density	kg m${}^{-3}$	1000
GOR	Gas/oil ratio	-	0.01
$P_{r}$	Reservoir pressure	N m ${}^{-2}$	15 × 10^6^
$P_{s}$	Separator pressure	N m${}^{-2}$	2 × 10^6^
$T_{a}$	Annulus temperature	K	301
$T_{w}$	Tubing temperature	K	305
$M_{w}$	Molar mass of gas	kg	0.028
*R*	Gas constant	J K${}^{-1}\;M^{-1}$	

**Table 4:** Hypothesis test for the residuals for estimates with the EKF, UKF and PF. √ indicates true hypothesis (which indicates the mean of the residual is zero) while X indicates the alternative hypothesis (which is that the mean of the residual is not zero). The *p*-values are also indicated.

Residual	EKF	UKF	PF
$P_{a}$	X $1.9\times 10^{-4}$	X $4.3\times 10^{-3}$	X $3.8\times 10^{-36}$
$P_{wh}$	X $1.5\times 10^{-3}$	X $3.2\times 10^{-12}$	X $2.2\times 10^{-58}$
$\rho_{t}$	√ $7.1\times 10^{-1}$	√ $6.6\times 10^{-1}$	X $3.9\times 10^{-3}$

**Table 5:** RMSE for estimates with the EKF, UKF and PF with casing heading instability. Hypothesis test on the residual is provided where √ indicates true hypothesis while X indicates the alternative hypothesis.

*u*	Residual	EKF	UKF	PF
0.60	$P_{a}$	X $9.1\times 10^{3}$	X 23	X $6.4\times 10^{4}$
	$P_{wh}$	X $7.8\times 10^{3}$	X $1.1\times 10^{2}$	X $5.0\times 10^{4}$
	$\rho_{t}$	√ 0.52	√ 6.4	X 6.2
0.75	$P_{a}$	X $1.4\times 10^{4}$	X 23	X $7.1\times 10^{4}$
	$P_{wh}$	X $7.9\times 10^{3}$	X $1.4\times 10^{2}$	X $3.7\times 10^{4}$
	$\rho_{t}$	√ 0.97	√ 6.6	X 6.3
0.90	$P_{a}$	X $2.6\times 10^{4}$	√ 25	X $3.0\times 10^{5}$
	$P_{wh}$	X $7.6\times 10^{3}$	X $1.2\times 10^{2}$	X $1.4\times 10^{5}$
	$\rho_{t}$	√ 2.1	√ 6.4	X 6.0

## Data Availability

Not applicable.
